# Demonstration of value of optimizing ECG triggering for cardiovascular magnetic resonance in patients with congenital heart disease

**DOI:** 10.1186/1532-429X-15-3

**Published:** 2013-01-16

**Authors:** Thomas Knesewitsch, Christian Meierhofer, Henrike Rieger, Jürgen Rößler, Michael Frank, Stefan Martinoff, John Hess, Heiko Stern, Sohrab Fratz

**Affiliations:** 1 Department of Pediatric Cardiology and Congenital Heart Disease, Deutsches Herzzentrum München, Klinikum an der Technischen Universität München, Lazarettstr. 36, 80636, Munich, Germany; 2 Siemens AG, Healthcare, Imaging & Therapy Systems, Magnetic Resonance, Erlangen, Germany; 3 Department of Radiology, Deutsches Herzzentrum München, Klinikum an der Technischen Universität München, Munich, Germany

**Keywords:** ECG triggering, Congenital heart disease, Blood flow quantification, Vectorcardiography (VCG)

## Abstract

**Background:**

Optimal ECG triggering is of paramount importance for correct blood flow quantification during cardiovascular magnetic resonance (CMR). However, optimal ECG triggering and therefore blood flow quantification is impaired in many patients with congenital heart disease (CHD) due to complex QRS patterns. Therefore, a new ECG-trigger algorithm was developed to address triggering problems due to complex QRS patterns.

The aim of this study was to test this new ECG-trigger algorithm in routine patients with CHD and its impact on blood flow quantification.

**Methods:**

35 consecutive routine patients with CHD undergoing CMR were included in the study. (40% Fallot’s Tetralogy, 20% aortic arch pathology, 14% transposition of the great arteries, 26% others; age 26+/−11 yrs).

In all patients, blood flow in the ascending aorta was quantified using the *old* ECG-trigger algorithm and the *new* ECG-trigger algorithm in random order. Blood flow quantified using the *old* or *new* ECG-trigger algorithm was compared by Bland-Altman analysis.

Three blinded investigators evaluated the vector clouds and trigger points of both ECG-trigger methods. Evaluation criteria were false positive and false negative triggered QRS complexes (specificity and sensitivity), and accuracy of detection. Accuracy of detection was defined as time scatter of the trigger around the correct trigger point.

**Results:**

Specificity, sensitivity, and accuracy of detection significantly increased using the *new* ECG-trigger algorithm compared to the *old* ECG-trigger algorithm.

Blood flow quantification using the *old* or *new* ECG-trigger algorithm differed more than 5% in 31% of the cases.

**Conclusions:**

Our results suggest that optimizing ECG triggering during CMR using our new algorithm can avoid errors of >5% in approximately 1/3 of routine patients with congenital heart disease (CHD). We furthermore suggest that incorrect ECG triggering appears to be problematic for blood flow quantification of many patients with CHD undergoing routine CMR.

## Background

Cardiovascular magnetic resonance (CMR) has evolved from a pure imaging method to a powerful tool in the diagnostic management of congenital heart disease (CHD) [1]. This evolution is in a large part due to the capability of CMR to measure flow by phase-velocity CMR [2]. To measure flow by phase-velocity CMR, synchronisation of the images to the heart phase is needed. This synchronisation is realised by ECG triggering. ECG triggering is based on real-time R-wave detection and ensures that each portion of an image is allocated to a specific phase of the cardiac cycle. Accordingly, an imprecise detection leads to an incorrect allocation of the images to the cardiac cycle and ultimately to false diagnostic results [2,3]. Therefore, optimal ECG triggering is of paramount importance for correct blood flow quantification during CMR.

Reasons for imprecise triggering are known and efficient algorithms have been developed and used for several years. An important reason beside the general noise in normal ECG is the MR-specific environment, especially the magnetohydrodynamic effect. This effect leads to a deformation in the ST segment and may exhibit a T-wave with a larger amplitude than the QRS complex and finally to a wrong triggering.

In normal ECGs these problems seem to have been solved satisfactorily [3].

However, optimal ECG triggering and therefore blood flow quantification are impaired in many patients with CHD due to complex QRS patterns.

For example patients with CHD such as Tetralogy of Fallot or Ebstein anomaly partially show complex ECGs [4,5]. Accordingly, in our routine clinical experience incorrect ECG triggering leading to unsatisfactory flow measurements using the old ECG-trigger algorithm occurs frequently. However, the exact extent of incorrect flow measurements is unknown. In a previous study 2 of 347 assessments even failed totally due to unreliable triggering [1].

Therefore, a new ECG-trigger algorithm was developed to address triggering problems and achieve better performance in patients with complex ECGs.

The new trigger is mainly based on a matched filter [6]. The matched filter is able to detect the R-waves on the rising edge of the R-wave after an initial learning phase.

The old trigger algorithm works with threshold values, which are derived from the ECG.

The aim of this study was to test this new ECG-trigger algorithm in routine clinical patients with CHD and to measure its impact on blood flow quantification.

## Methods

### Study population

35 consecutive routine patients with CHD undergoing CMR were included in the study. Fourteen patients had Fallot’s Tetralogy, seven patients aortic arch pathology, five patients atrial switch operation for transposition of the great arteries, and nine patients had other complex congenital heart disease.

The patients consisted of 11 females and 24 males. The average age was 26 years +/−11 years ranging from 12 to 60 years.

### Phase-velocity CMR measurements

For cardiovascular magnetic resonance (CMR) a standard cardiac 1.5 Tesla CMR scanner and a standard cardiac 12-channel coil (MAGNETOM Avanto®, software version VB15, Siemens Healthcare, Erlangen, Germany) was used.

To compare the difference of both triggering methods in all patients, the blood flow in the ascending aorta was quantified using the *old* ECG-trigger algorithm and the *new* ECG-trigger algorithm in random order directly after each other. The slice orientation and all other acquisition parameters were not changed between the two phase-velocity CMR measurements: free breathing (typical acquisition times: around 3 minutes), retrospective ECG gating, the velocity encoding was set to 200–550 cm/s according to the assumed jet velocity, the slice thickness was 5 mm, repetition time 36.7 ms, echo time 3.09 ms, flip angle 30°, averages 3, segmentation 3, number of phase-encoding steps 192, receiver bandwidth 31.25 kHz, rectangular field of view 260 to 330 × 330 mm, matrix 256 × 256, phase partial Fourier off. Data was reconstructed to provide 30 magnitude (anatomic) and phase (velocity-mapped) images per cardiac cycle. All measurements were automatically compensated for the concomitant gradient effects. It is important to note, that we always imaged the vessel’s region of interest at isocenter to maximize gradient fidelity. Additionally, we took meticulous care of placing the region of interest into the center of the imaging plane. The region of interest was placed into the center of the imaging plane because placing the region of interest out of the center of the imaging plane can lead to large errors [7].

Furthermore, the ECG was monitored during acquisition. The running acquisition was always aborted when extra systoles were noted.

The phase contrast images were processed with the post-processing software ARGUS (syngo MultiModality Workplace, version VE23B, Siemens Healthcare, Erlangen, Germany) by using manual contour segmentation in all phases of the cardiac cycle.

### ECG-triggering methods

#### *old ECG-trigger algorithm*

The *old* algorithm is based on two ECG channels acquired simultaneously. The signals are supplied in a first processing branch to a low-pass filter and a derived value sum generator. The output signal compared with a threshold value generates a first comparison result. Additionally, the signals are fed in a second processing branch to a derived value generator. The output signal compared with an upper and lower threshold generates a second comparison result. Those first and second branches exist for both ECG channels. All first and second comparison results are evaluated in a weighted logic circuit, whose output are triggers for the MRI measurement [8]. The mentioned upper and lower threshold values are learned during the initial learning phase.

#### *new ECG-trigger algorithm*

The *new* algorithm learns the shape of the rising edge of the R-wave during the initial learning phase in both ECG channels. Then, the trigger algorithm continuously compares the incoming ECG signal with the learned shape by realtime processing [6,9]. The comparison is based on the following means: A matched filter which is widely used in telecommunications [10] and a filter that sums up the squared differences between the incoming ECG signal and the learned shape. The latter corresponds to a pattern-matching mechanism. Additionally, the angle of the VCG vector which is spanned by the two ECG channels at each time instant is utilized for detection of the R-wave. This angle criterion is used as a necessary condition but not as a sufficient condition for the generation of triggers.

### ECG analysis

The aim of the analysis was to detect failed triggering of the QRS complex and to evaluate the accuracy of the triggering. Both failures will probably lead first to a higher jitter of the RR interval and second to a false flow calculation.

For the evaluation of the triggering the ECG was recorded from the beginning to the end of each sequence, totalling in 192 QRS complexes, respectively.

The ECG data was analysed in two steps:

1. Failed triggering

**Figure 1:**
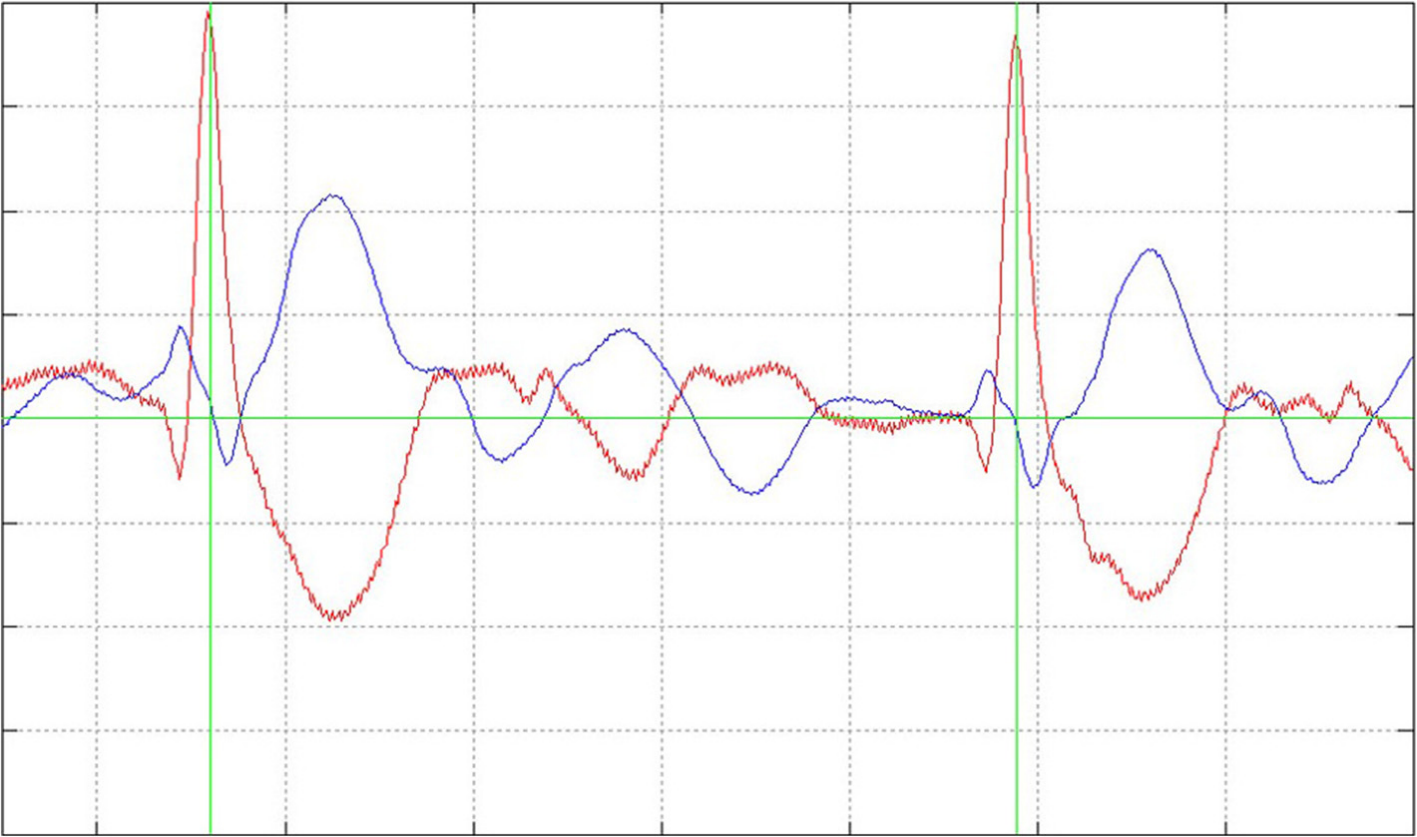
**Correct ECG-triggering. **Detail view of two typical beats of the Two-Channel ECG (Channel 1 red, Channel 2 blue) with the green trigger-lines. Every beat of the entire ECG recording of each patient was checked for missed or redundant triggering.

**Figure 2:**
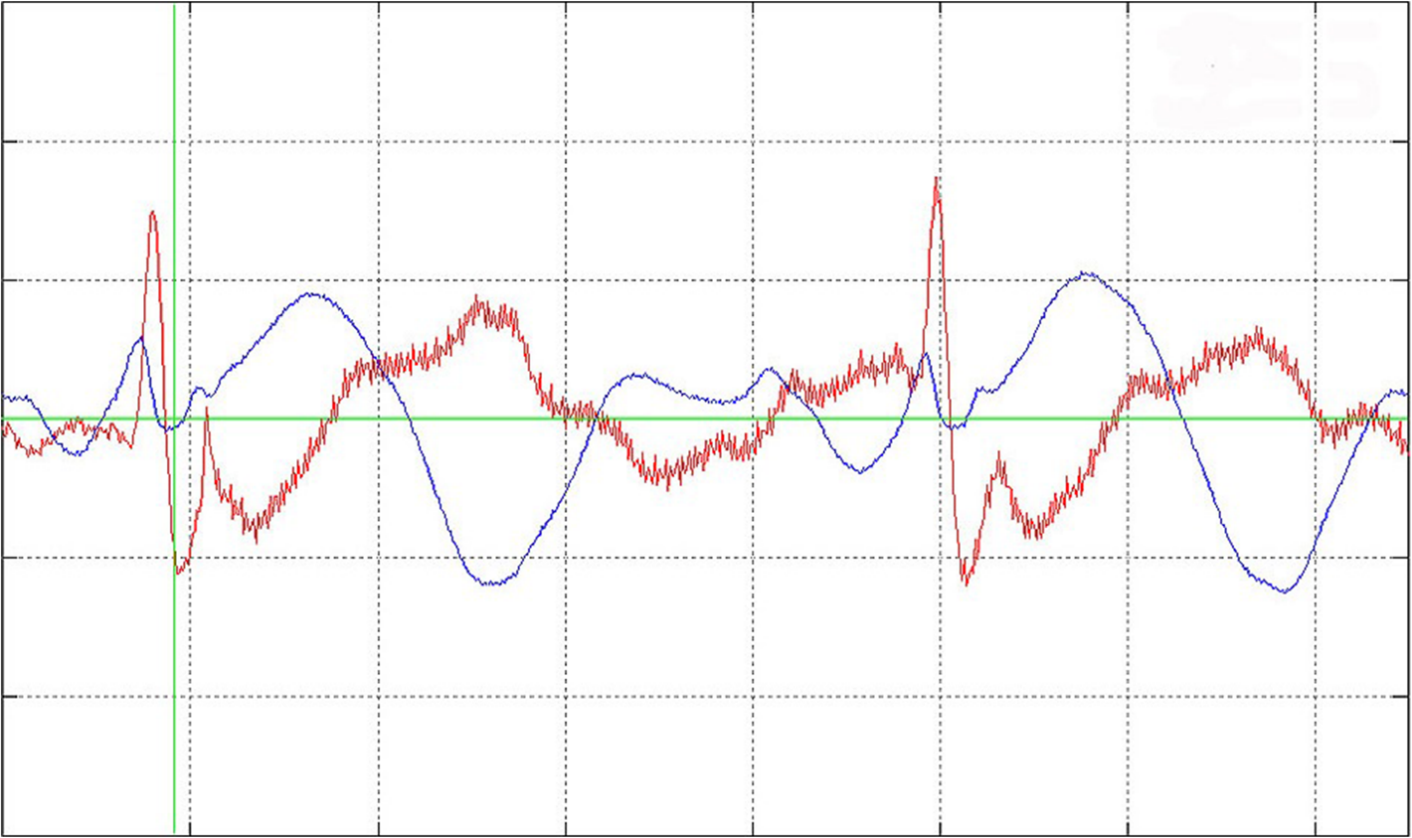
**False negative ECG-triggering. **Detailed view of a false negative ECG triggering. The second ECG beat was not detected.

**Figure 3:**
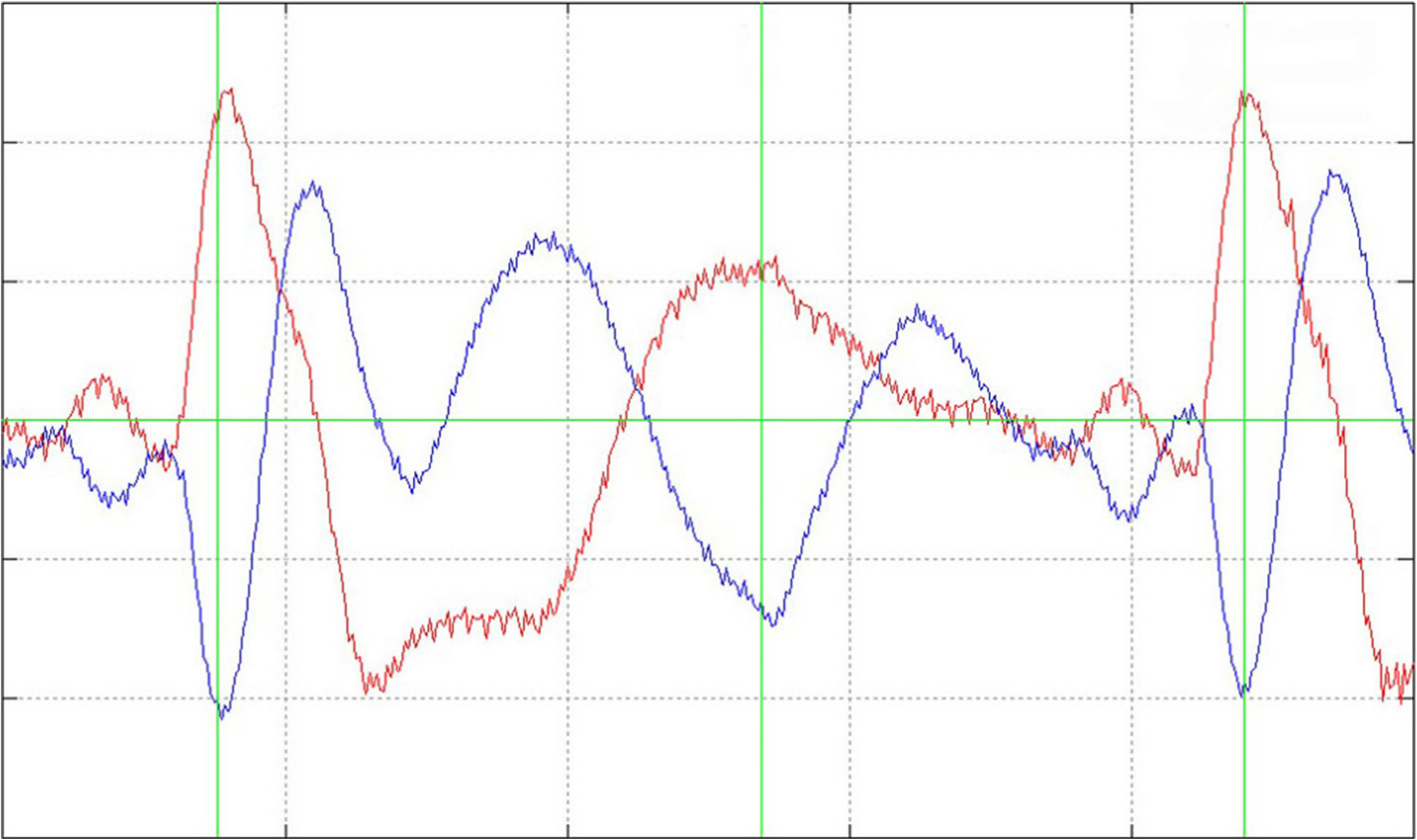
**False positive ECG-triggering. **Detailed view of a false positive ECG triggering. An additional trigger is set without a correspondent ECG beat between two correctly triggered ECG beats.

A two-channel ECG with the trigger marking was computed to analyse manually that exactly one trigger point is dedicated to every QRS complex. A heart cycle with two trigger points was counted as a false positive and a not triggered QRS-complex as a false negative (see Figures 1, 2 and 3). The results were used to calculate Sensitivity, Specificity and Performance Index (PI) of the method according to the following equations:

$$\begin{array}{l} \mathrm{Sensitivity}=\left(\mathrm{number}\;\mathrm{of}\;R\;\mathrm{peaks}-\mathrm{number}\;\mathrm{of}\;\mathrm{false}\;\mathrm{negatives}\right)\\ \;/\mathrm{number}\;\mathrm{of}\;R\;\mathrm{peaks} \end{array}$$

$$\begin{array}{l} \mathrm{Specificity}=\left(\mathrm{number}\;\mathrm{of}\;R\;\mathrm{peaks}-\mathrm{number}\;\mathrm{of}\;\mathrm{false}\;\mathrm{positives}\right)\\ \;/\mathrm{number}\;\mathrm{of}\;R\;\mathrm{peaks}\; \end{array}$$

$$\begin{array}{l} \mathrm{Performance}\;\mathrm{Index}=[(\mathrm{number}\;\mathrm{of}\;R\;\mathrm{peaks}\\ \;-(\mathrm{number}\;\mathrm{of}\;\mathrm{false}\;\mathrm{negatives}\\ \;+\mathrm{number}\;\mathrm{of}\;\mathrm{false}\;\mathrm{positives})]\\ \;/\mathrm{number}\;\mathrm{of}\;R\;\mathrm{peaks} \end{array}$$

2. Accuracy of triggering

**Figure 4:**
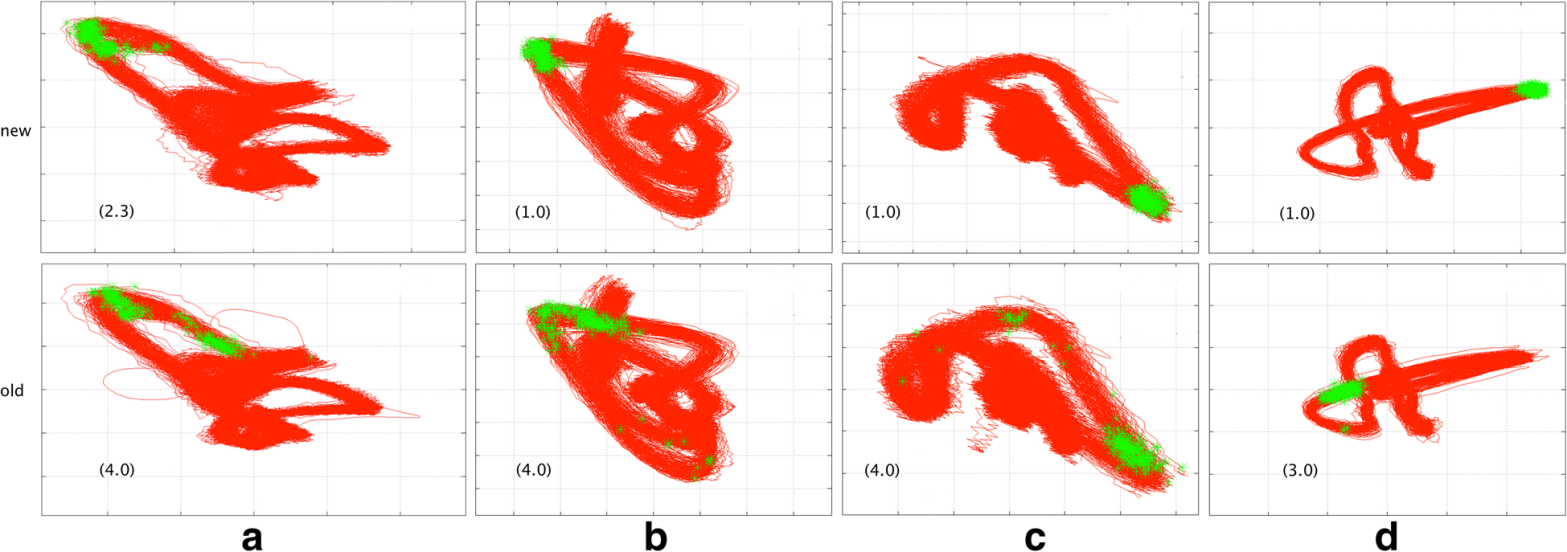
**Examples of vector clouds. **Vector clouds of four patients (**a**-**d**), the green points are trigger-points, which optimally should be located in the R-loop (like in upper row d). Upper row: *new* algorithm, lower row: *old* algorithm. In parenthesis the average grading of the three blinded graders from 1 to 4.

A good accuracy of the triggering means that every trigger point is located at the peak of the R-wave or at least always on the same one point of the R-wave. Bad triggering means that the trigger points are scattered over the ascending and descending part of the R-wave or even in a different part of the ECG, e.g. trigger points in the T-wave instead of the R-wave due to the magnetohydrodynamic effect (see Figure 4). To estimate this kind of error we decided to evaluate the triggering with the help of vectorcardiography (VCG). VCG is a 2D illustration of the electric conduction of the heart. In analogy to the ECG, the VCG consists of a P-, T- and an R-loop. The advantage of this method is that all 192 heart phases and their triggering can be plotted as a vector cloud in one single image (Figure 4). The clouds were evaluated in a blinded fashion as pairs in random order from three different investigators (CM, HS, SF), according to a predefined semiquantitive subjective grading of the ECG-triggering (1: “very good”, small scatter around the same area of the QRS-loop; 2: “good”, large scatter around the same area of the QRS-loop; 3: “bad”, < 5/192 (3%) trigger points in a different area of the QRS-loop; 4: “bad”, ≥ 5/192 (3%) trigger points in a different area of the QRS-loop).

3. Flow quantification

The results of the measurement of the *old* and *new* ECG-trigger algorithm were compared by Bland-Altman analysis [11]. The confidence interval was set to the mean value +/−1.96 of the standard deviation. Additional percentage lines were drawn to illustrate measurements with more than 5% and 10% difference.

4. Correlations between the changes in standard deviation, flow and accuracy

The standard deviation (SD) of the RR interval is a potential marker for unreliable triggering. Failed triggering will obviously lead to a higher SD. And probably a lower accuracy will also lead to a higher SD. Therefore a difference in SD between both measurements should be correlated with the power of the triggering method. We also expect a correlation between the SD and differences in the flow.

To compare these values, the differences in the SD and net flow were calculated as percentage of the mean value SD [%] and net flow [%].

## Results

### ECG analysis

#### *Failed triggering*

The analysis of the wrong set triggers showed that the new trigger algorithm had a lower rate of wrong ECG triggering (see Table 1).

**Table 1:** Results of sensitivity, specificity and performance

	**False positives**	**False negatives**	**Specificity (%)**	**Sensitivity (%)**	**Performance index (%)**
**old algorithm (N = 6811)**	22 *	63	99.68	99.08	98.75
**new algorithm (N = 6802)**	0	16	100.00 **	99.76 ***	99.76

Specificity, sensitivity and Performance Index of detection increased using the *new* ECG-trigger algorithm compared to the *old* ECG-trigger algorithm.

* 22 false positive trigger points in three of 35 patients.

** p = 0.104 vs. old algorithm.

*** p = 0.043 vs. old algorithm.

#### *Accuracy of triggering*

Triggering was found to be more accurate using the *new* algorithm: Average grading for the *old* algorithm was 3.2 +/−1.0, average grading for the *new* algorithm was 1.5 +/−0.6 (p < 0.001). In 18 (51%) cases the accuracy of the new algorithm was remarkably better than the old (with a difference of grading of more than 2) and in 16 (46%) cases slightly better or equal (less than 2,0 difference). Only in one case, the accuracy of the old algorithm seemed to be better.

#### *Flow quantification*

**Figure 5:**
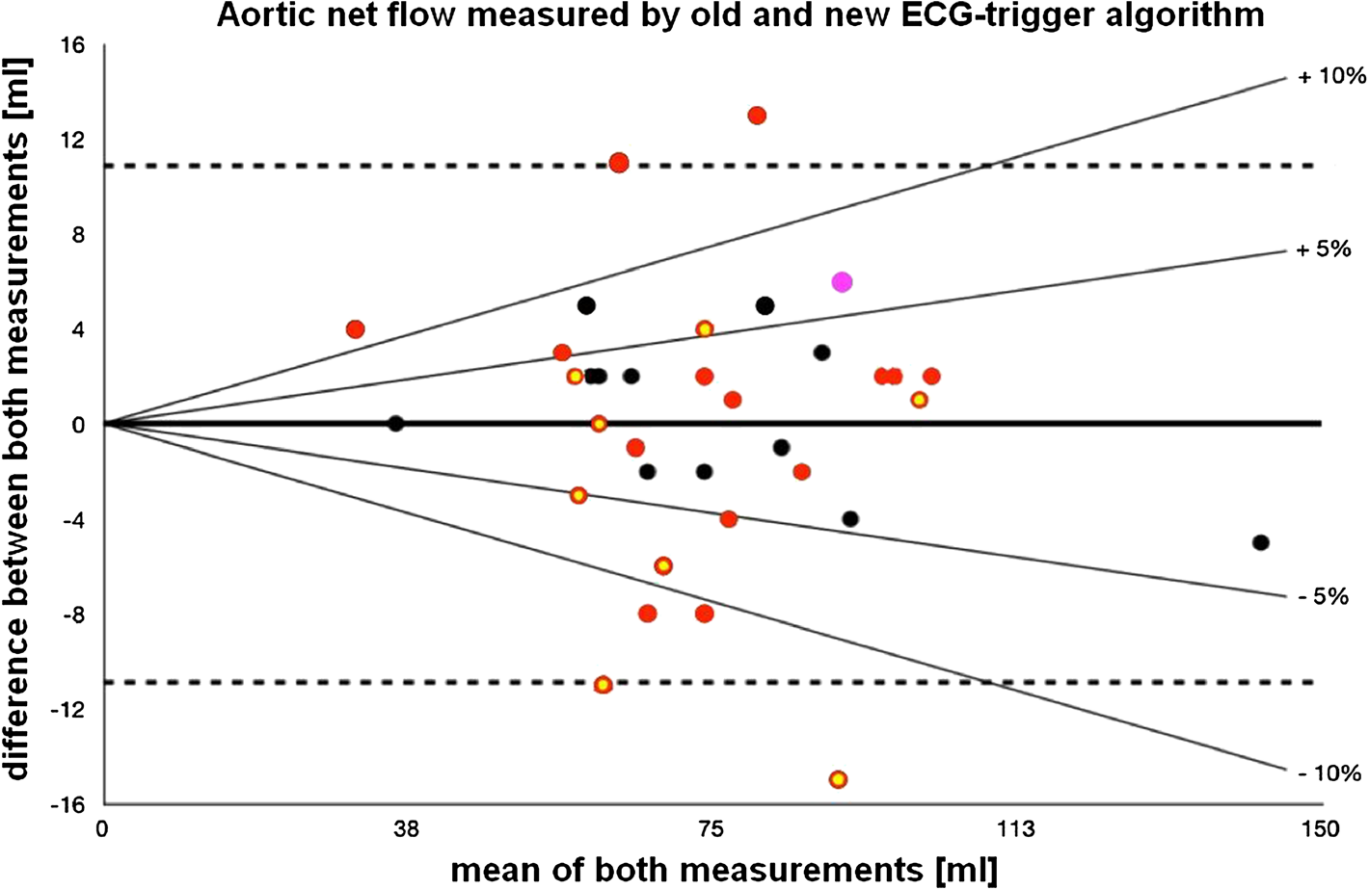
**Modified Bland-Altman analysis. **Bland-Altman [11] graph showing the difference of measured Aortic flow between the *old* and *new* ECG-trigger algorithm (mean difference/bold continuous line: 0.0 ml; upper and lower limits of agreement/dashed lines: ± 10.9 ml). The Bland-Altman graph has been modified by adding lines depicting the 5% and 10% difference of the mean Aortic flow. Red dots: “bad *old* ECG trigger cases”, specifically cases in which the average grading of the *old* ECG trigger algorithm was graded as worse than “bad”(trigger points in a different area of the QRS-loop). Red/yellow dots: “bad *old* ECG trigger cases”, specifically cases in which both the average grading of the *old* ECG trigger algorithm was worse than “bad”(trigger points in a different area of the QRS-loop) and that had > 2 false positive or false negative triggered QRS-complexes. Violet dot: “bad *new* ECG trigger case”, specifically only case in which the average grading of the *new* ECG trigger algorithm was graded worse than “bad”(trigger points in a different area of the QRS-loop). Black dots: “good *old* and *new* ECG trigger case”, note that all these cases lie within the 10% difference lines, depicting good agreement between measured Aortic flow using the *old* and *new* ECG-trigger algorithm.

Measurements of aortic flow using the two triggering methods showed a range of differences (Figure 5). In 11 (31%) cases, the flow measurements differed by +/− 5% and 7 (20%) of these cases showed a difference of more than +/−10% with a maximum of 18%.

#### *Correlations between the changes in SD, net flow and accuracy*

The SD [%] differed between both algorithms (p = 0.028). The differences of SD [%] also correlated with differences in net flow [%] (p = 0.027 r = 0.373).

Furthermore, differences in grading as a marker for the accuracy correlated with differences in the SD [%] (p = 0.005 r = 0,462).

## Discussion

Due to the positive correlation between our appraisal of the accuracy and the difference in the flow quantifications we can assume that inefficient triggering has a considerable impact on the quality of flow quantification. However, other concomitant factors, which affect the flow in the ascending aorta, cannot totally be eliminated by our method. It is also difficult to achieve objective parameters for the evaluation of the accuracy. The obviously slightly better performance index, mainly based on missed QRS complexes, cannot statistically be correlated with the flow due to the very good performance index of both methods. Therefore, it is not possible to trace a specific difference in flow quantification totally back to an inefficient triggering. Nevertheless, we can say that the new algorithm works better under the same conditions than the old algorithm and because of that we can expect better results.

## Conclusions

The difference in the flow quantification underlines the supposed complications. If we presume that a difference of more than 5% between two measurements probably leads to a clinically relevant misinterpretation and that a physical change in aortic flow under stable patient conditions can be neglected, 1/3 of the assessments probably fail due to incorrect measurements. The study shows that remarkable differences between the new and the old algorithms exist. The new algorithm showed significantly better triggering in all tested parameters. We can assume that these differences in triggering are an important source of error in the blood flow quantification. The new algorithm will probably help to achieve better results in many patients with CHD undergoing CMR.

## Competing interests

JR and MF are employees of Siemens AG, Magnetic Resonance, and have patents [6,9] on the new ECG-trigger algorithm. The other authors declare that they have no competing interests.

## Authors’ contributions

TK took part in the design of the study, analyzed and interpreted the entire data, drafted the first version and revised several drafts of the manuscript. CM took part in the conception and design of the study, analyzed and interpreted parts of the entire data, and reviewed the drafts of the manuscript for important intellectual content. HR took part in the conception and design of the study, and reviewed the drafts of the manuscript for important intellectual content. JR has patents on the new ECG-trigger algorithm, took part in the conception and design of the study, and reviewed the drafts of the manuscript for important intellectual content. MF has patents on the new ECG-trigger algorithm, took part in the conception and design of the study, and reviewed the drafts of the manuscript for important intellectual content. SM took part in the conception and design of the study, reviewed the drafts of the manuscript for important intellectual content. JH took part in the conception and design of the study, reviewed the drafts of the manuscript for important intellectual content. HS took part in the conception and design of the study, analyzed and interpreted parts of the entire data, reviewed the drafts of the manuscript for important intellectual content. SF planned and designed the study, carried out most CMR studies, analyzed and interpreted the entire data, and revised several drafts of the manuscript. All authors read and approved the final manuscript.
